# Supplementary material for: A meta-analysis of infant gut viromes reveals global patterns in bacteriophage community assembly and functional capacity over the first three years of life
Source: Nat Commun. 2026 Jun 18;17:7698. doi: 10.1038/s41467-026-74609-5 (PMC13434738; doi:10.1038/s41467-026-74609-5)
Supplement: Supplementary file 1 [file 41467_2026_74609_MOESM1_ESM.pdf]

Supplementary Figures

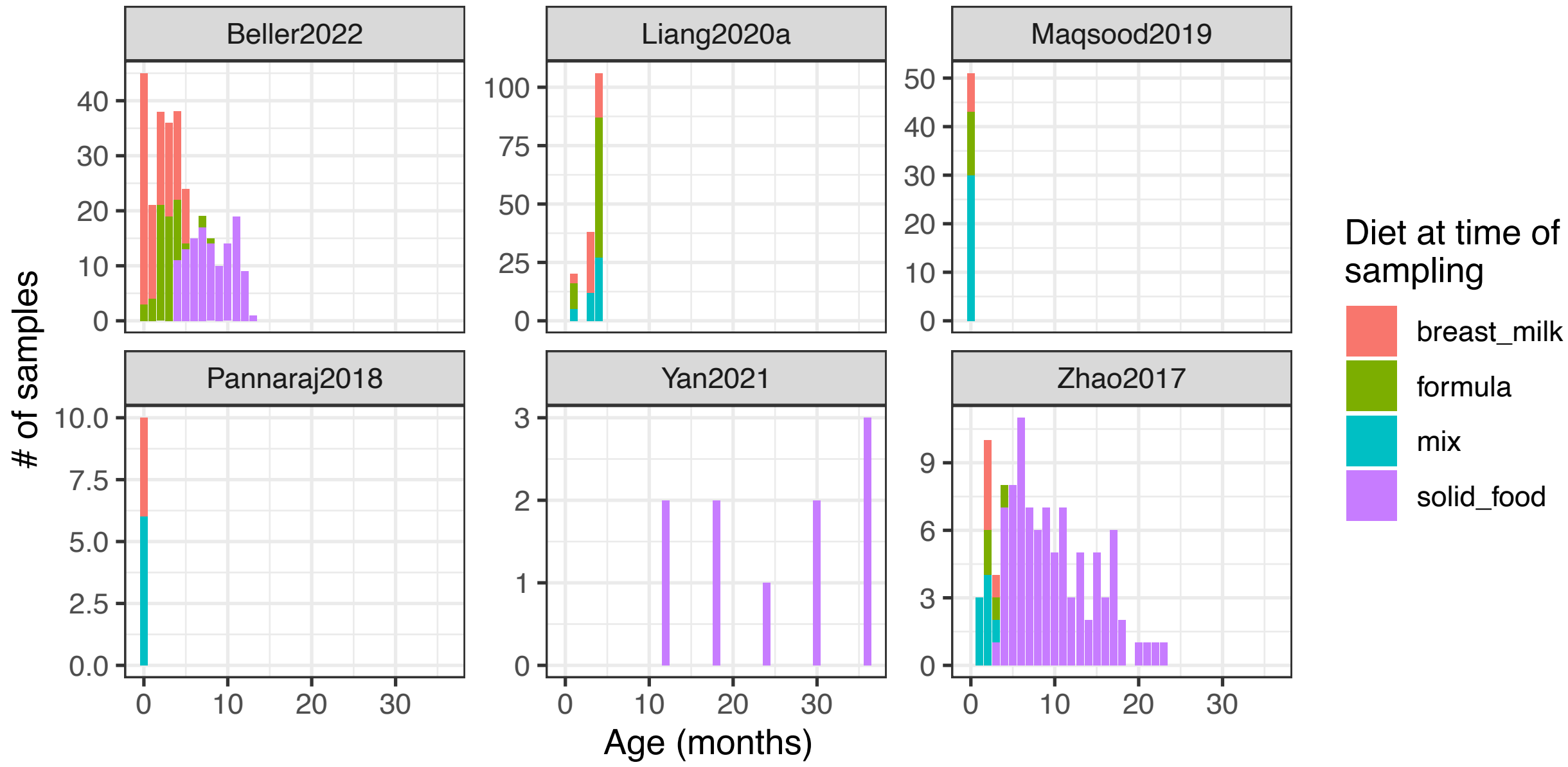

Supplementary Figure 1. Overview of infant diet metadata across six cohorts. Distribution of infant diet at time of sampling for studies with available metadata (n = 645 samples from 6 studies). “Mix” refers to a combination of breast milk and formula, “solid_food” indicates the consumption of solids, either complementary to breast milk/formula, or exclusively.

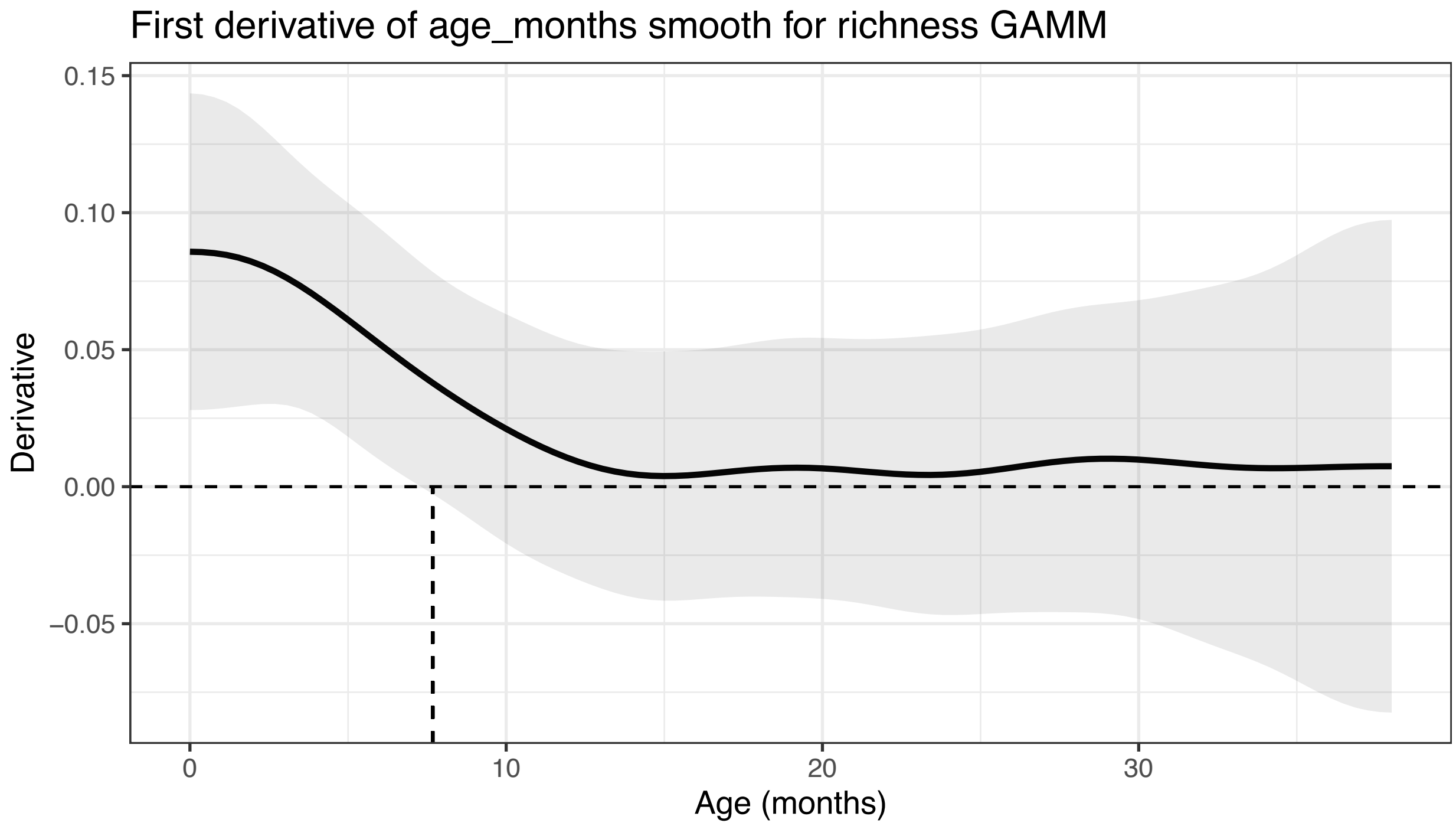

Supplementary Figure 2. Rate of change of gut viral richness during early life. The first derivative of the generalized additive mixed model (GAMM) for observed richness (see Figure 2C) was calculated with respect to infant age in months. The solid line corresponds to the mean derivative, and the shaded ribbon represents the 95% confidence interval. The vertical dashed line at x = 7.68 months indicates the earliest point at which the confidence interval crosses y = 0. This threshold represents the point at which the rate of richness accumulation is no longer significantly different from zero, indicating that virome richness has plateaued.

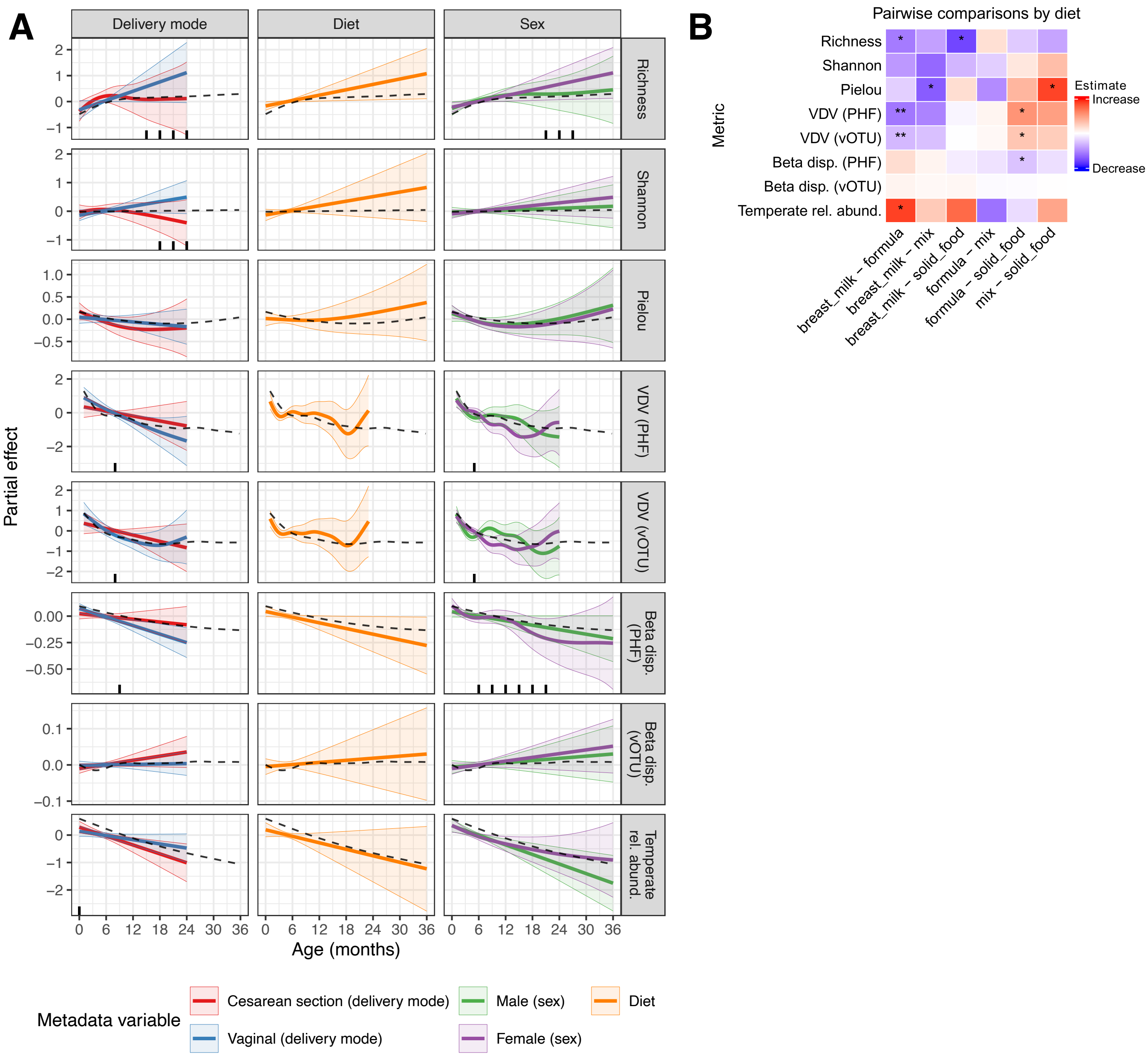

Supplementary Figure 3. Subset-modelling approach to evaluate the effects of sex, delivery mode, and diet on gut virome development. (A) Generalized additive mixed models (GAMMs) were fit to subsets of the overall 1,893 samples according to metadata available: infant sex (n = 774 with metadata), delivery mode (n = 500), or diet (n = 645). The fitted smooths represent the mean of the non-linear relationships between the metric under study (richness, Pielou's evenness, Shannon index, virome developmental velocity (VDV), beta dispersion, or temperate phage relative abundance) and infant age in months, and the shaded ribbon represents the 95% confidence interval. Dotted lines represent the fitted smooths of the original GAMMs (from main text & figures) where sex, delivery mode, and diet were not included in the models. Vertical bars adjacent the x-axis denote timepoints with a significant difference ($p < 0.05$) between male/female or vaginal/cesarean trajectories. Model formula which includes sex metadata: RESPONSE ~ s(age_months, by = sex) + sex + log10(depth) +

s(age_months, study, bs = "fs", m = 1) + s(subjectID, bs = "re"). Model formula which includes delivery mode metadata: RESPONSE ~ s(age_months, by = delivery_mode) + delivery_mode + log10(depth) + s(age_months, study, bs = "fs", m = 1) + s(subjectID, bs = "re"). Model formula which includes diet metadata: RESPONSE ~ s(age_months) + diet + log10(depth) + s(age_months, study, bs = "fs", m = 1) + s(subjectID, bs = "re"). **(B)** Summary of pairwise dietary contrasts from emmeans. Cell colour reflects estimated effect size on the link scale, and asterisks indicate significance after correcting for multiple comparisons (* p < 0.05, ** p < 0.01).

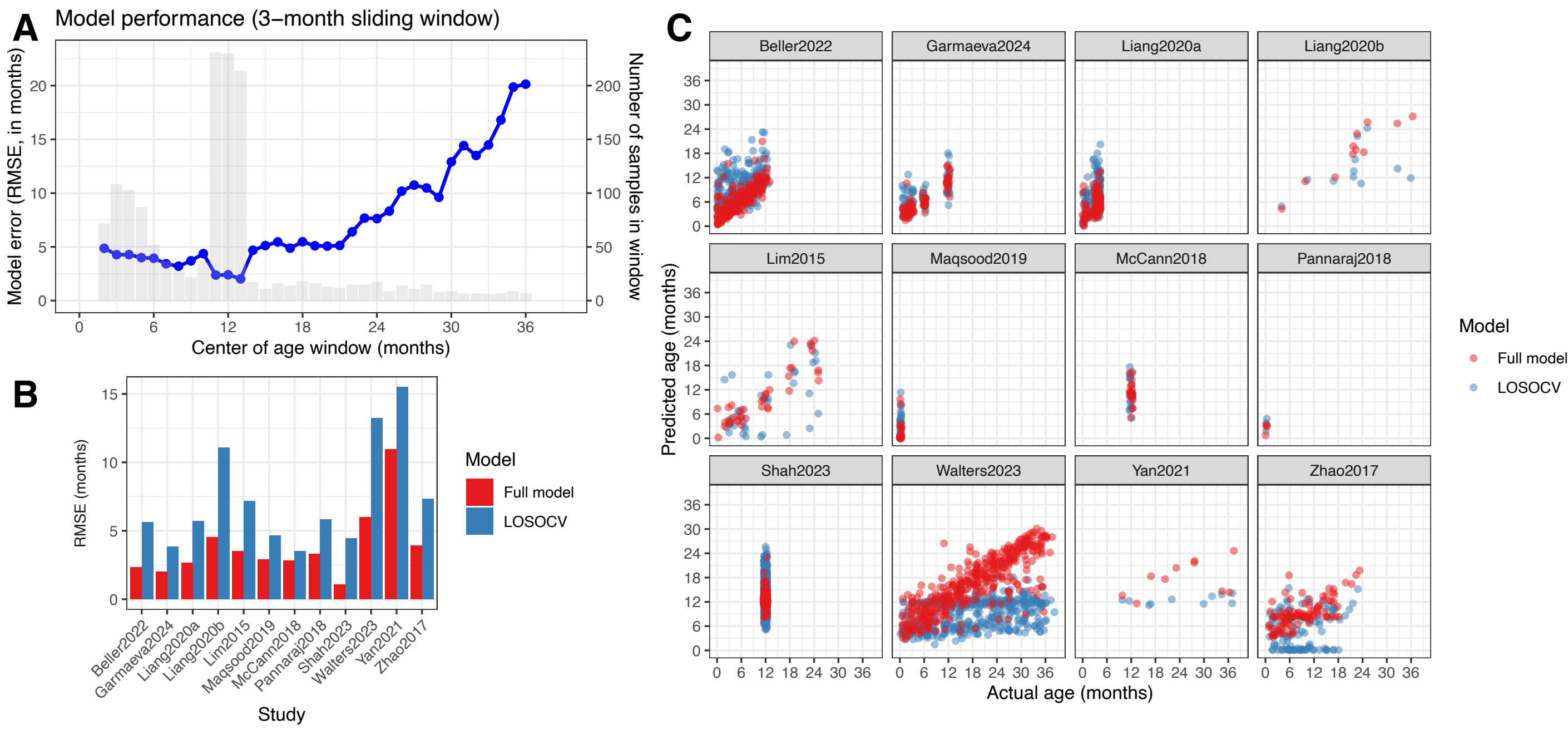

Supplementary Figure 4. Validation of the age predictor model. (A) Model performance throughout development, evaluated as the root mean square error (RMSE) within 3-month sliding windows. Points indicate the RMSE for each window, while bars indicate the number of sample size (n) within that 3-month period. **(B)** Comparison of model accuracy across the 12 cohorts evaluated via leave-one-study-out cross-validation (LOSOCV). Red bars (full model) represent the RMSE when the model trained on the entire original training dataset is tested on a specific cohort. Blue bars (LOSOCV) represent the RMSE when the model is trained excluding that specific cohort and then tested on it. **(C)** Age predictions from both the full regression model and LOSOCV model permutations, colour-coded by model.

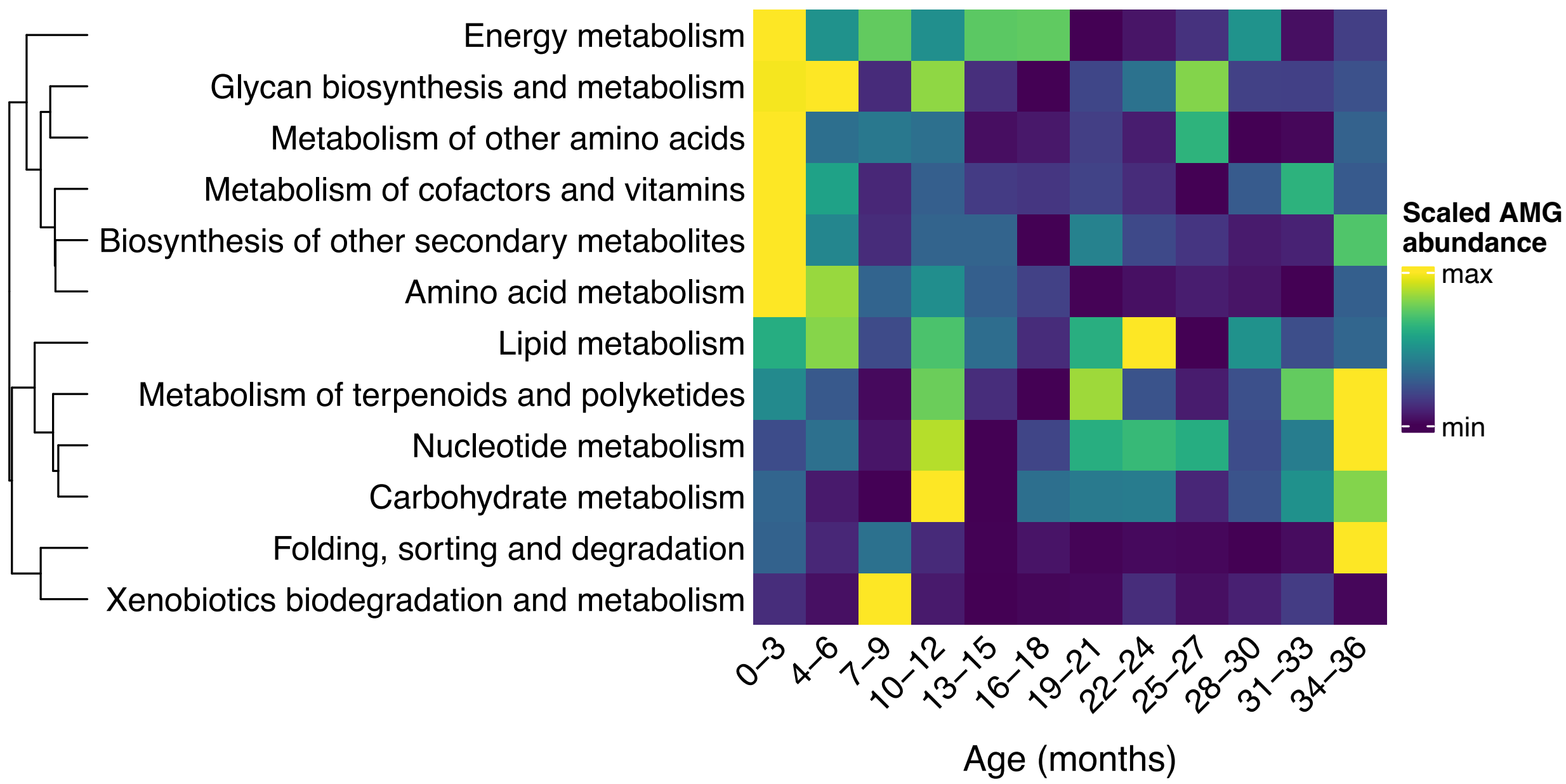

Supplementary Figure 5. Functional maturation of the infant gut virome. Heatmap of the mean relative abundance of KEGG metabolism categories across 3-month age bins. To highlight successional shifts, values are min/max scaled across each category (row).

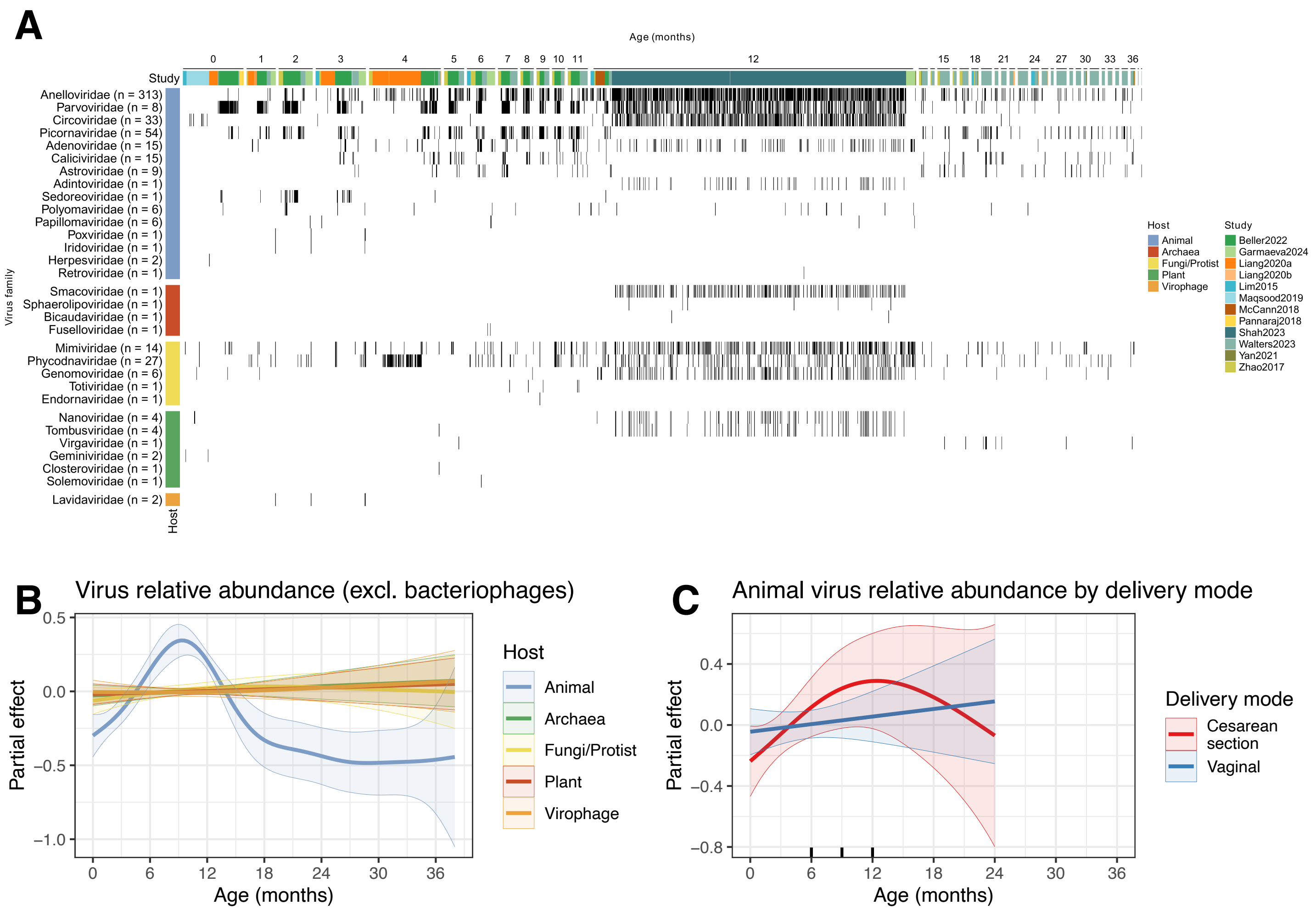

Supplementary Figure 6. Prevalence and abundance of non-bacteriophage viruses in the infant gut. (A) Heatmap of presence (black) and absence (white) of 31 non-bacteriophage viral families across infant viromes, grouped by viral host (rows) and infant age in months (columns). **(B)** A generalized additive mixed model (GAMM) was fit to model relative abundance of non-bacteriophage viruses among all infants. The fitted smooths represent the mean of the non-linear relationships between the cumulative relative abundance of non-bacteriophage viruses (grouped by viral host) and infant age in months, and the shaded ribbon represents the 95% confidence interval. Fitted smooths are colour-coded by viral host. Only animal virus abundances were significantly associated with age ($p < 0.001$). Model formula: relative.abundance ~ s(age_months, by = host) + host + log10(depth) + s(age_months, study, bs = "fs", m = 1) + s(subjectID, bs = "re"). **(C)** A generalized additive mixed model (GAMM) was fit to model relative abundance of animal viruses by delivery mode in a subset of the overall 1,893 samples with delivery mode metadata available (n = 500 samples). The fitted smooths represent the mean of the non-linear relationships between the cumulative relative abundance of animal viruses and infant age in months, and the shaded ribbon represents the 95% confidence interval. Fitted smooths are colour-coded by delivery mode. Vertical bars adjacent the x-axis denote timepoints

with a significant difference ($p < 0.05$) between vaginal/cesarean trajectories, as determined by emmeans. Model formula: animal.virus.relative.abundance ~ s(age_months, by = delivery_mode) + delivery_mode+ log10(depth) + s(age_months, study, bs = "fs", m = 1) + s(subjectID, bs = "re").

PRISMA 2020 flow diagram

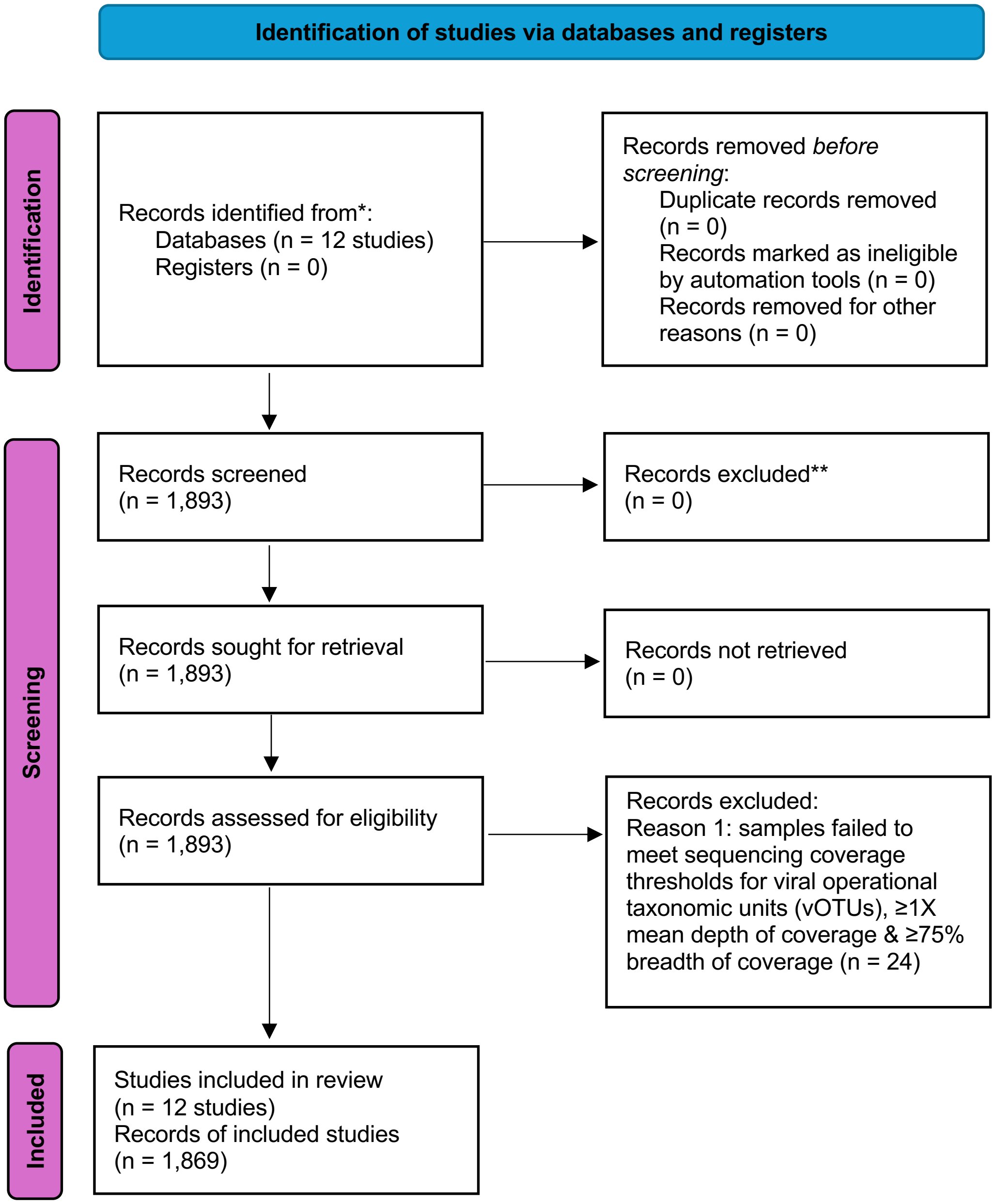

Source: Page MJ, et al. BMJ 2021;372:n71. doi: 10.1136/bmj.n71.

This work is licensed under CC BY 4.0. To view a copy of this license, visit https://creativecommons.org/licenses/by/4.0/
