# Supplementary material for: A meta-analysis of infant gut viromes reveals global patterns in bacteriophage community assembly and functional capacity over the first three years of life
Source: Nat Commun. 2026 Jun 18;17:7698. doi: 10.1038/s41467-026-74609-5 (PMC13434738; doi:10.1038/s41467-026-74609-5)
Supplement: Supplementary file 2 [file 41467_2026_74609_MOESM2_ESM.pdf]

Description of Additional Supplementary Files

File Name: Supplementary Data 1

Description: Summary of metadata and sampling information for the 12 integrated cohorts. Overview of the studies included in this meta-analysis, including sampling location, cohort size (number of participants & number of samples), and the age range of sampling, sequencing approach & depth per sample, and available metadata. The study design for each study is abbreviated as follows: L (longitudinal) or C (cross-sectional).
