# Supplementary material for: A meta-analysis of infant gut viromes reveals global patterns in bacteriophage community assembly and functional capacity over the first three years of life
Source: Nat Commun. 2026 Jun 18;17:7698. doi: 10.1038/s41467-026-74609-5 (PMC13434738; doi:10.1038/s41467-026-74609-5)
Supplement: Supplementary file 3 [file 41467_2026_74609_MOESM4_ESM.pdf]

nature portfolio

Corresponding author(s): Shamash M, Maurice CF

Last updated by author(s): Jun 2, 2026

Reporting Summary

Nature Portfolio wishes to improve the reproducibility of the work that we publish. This form provides structure for consistency and transparency in reporting. For further information on Nature Portfolio policies, see our Editorial Policies and the Editorial Policy Checklist.

Statistics

For all statistical analyses, confirm that the following items are present in the figure legend, table legend, main text, or Methods section.

n/a	Confirmed	
☐	☒	The exact sample size (*n*) for each experimental group/condition, given as a discrete number and unit of measurement
☒	☐	A statement on whether measurements were taken from distinct samples or whether the same sample was measured repeatedly
☐	☒	The statistical test(s) used AND whether they are one- or two-sided<br>*Only common tests should be described solely by name; describe more complex techniques in the Methods section.*
☐	☒	A description of all covariates tested
☐	☒	A description of any assumptions or corrections, such as tests of normality and adjustment for multiple comparisons
☐	☒	A full description of the statistical parameters including central tendency (e.g. means) or other basic estimates (e.g. regression coefficient) AND variation (e.g. standard deviation) or associated estimates of uncertainty (e.g. confidence intervals)
☐	☒	For null hypothesis testing, the test statistic (e.g. *F*, *t*, *r*) with confidence intervals, effect sizes, degrees of freedom and *P* value noted<br>*Give P values as exact values whenever suitable.*
☒	☐	For Bayesian analysis, information on the choice of priors and Markov chain Monte Carlo settings
☒	☐	For hierarchical and complex designs, identification of the appropriate level for tests and full reporting of outcomes
☐	☒	Estimates of effect sizes (e.g. Cohen's *d*, Pearson's *r*), indicating how they were calculated

Our web collection on statistics for biologists contains articles on many of the points above.

Software and code

Policy information about availability of computer code

Data collection	NCBI Sequence Read Archive, European Nucleotide Archive, European Genome-Phenome Archive, KOfam (v20250201), GTDB database (release 202)
Data analysis	The following tools/libraries were used in this study: fastp (v0.23.4), bowtie2 (v2.5.2), metaSPAdes (v3.15.4), geNomad (v1.7.4), BLASTN (v2.14.0), iPHoP (v1.0.0), BACPHLIP (v0.9.6), R (v4.5.1), phyloseq (1.52.0), mgcv (v1.9-3), gratia (v0.11.1), emmeans (v2.0.0), vegan (2.7-2), randomForest (v4.7-1.2), caret (v7.0-1), ComplexHeatmap (v2.24.1), prodigal-gv (v2.11.0), mmseqs2, HMMER (v3.4), sf (v1.0-21), rnaturalearth (v1.1.0), rhmmer (v0.2.0), samtools (v1.18).<br>Code used for data analysis is available at https://zenodo.org/records/19701266 .

For manuscripts utilizing custom algorithms or software that are central to the research but not yet described in published literature, software must be made available to editors and reviewers. We strongly encourage code deposition in a community repository (e.g. GitHub). See the Nature Portfolio guidelines for submitting code & software for further information.

Data

Policy information about availability of data

All manuscripts must include a data availability statement. This statement should provide the following information, where applicable:

- Accession codes, unique identifiers, or web links for publicly available datasets
- A description of any restrictions on data availability
- For clinical datasets or third party data, please ensure that the statement adheres to our policy

The raw virome sequencing data for each study are available in the NCBI Sequence Read Archive (SRA) under the following BioProject accessions: Lim 2015 (PRJNA284162), Zhao 2017 (PRJNA387903), McCann 2018 (PRJNA385126), Maqsood 2019 (PRJEB33578), Liang 2020a (PRJNA524703), Beller 2022 (PRJNA693793), Walters 2023 (PRJNA916952), Shah 2023 (PRJEB46943), Pannaraj 2018 (PRJNA427187), Yan 2021 (PRJNA641593), Liang 2020b (PRJNA524703). The raw virome sequencing data from Garmaeva 2024 study were not publicly available and were made available upon request from the study authors.

Research involving human participants, their data, or biological material

Policy information about studies with human participants or human data. See also policy information about sex, gender (identity/presentation), and sexual orientation and race, ethnicity and racism.

Reporting on sex and gender	*Use the terms sex (biological attribute) and gender (shaped by social and cultural circumstances) carefully in order to avoid confusing both terms. Indicate if findings apply to only one sex or gender; describe whether sex and gender were considered in study design; whether sex and/or gender was determined based on self-reporting or assigned and methods used.* *Provide in the source data disaggregated sex and gender data, where this information has been collected, and if consent has been obtained for sharing of individual-level data; provide overall numbers in this Reporting Summary. Please state if this information has not been collected.* *Report sex- and gender-based analyses where performed, justify reasons for lack of sex- and gender-based analysis.*
Reporting on race, ethnicity, or other socially relevant groupings	*Please specify the socially constructed or socially relevant categorization variable(s) used in your manuscript and explain why they were used. Please note that such variables should not be used as proxies for other socially constructed/relevant variables (for example, race or ethnicity should not be used as a proxy for socioeconomic status).* *Provide clear definitions of the relevant terms used, how they were provided (by the participants/respondents, the researchers, or third parties), and the method(s) used to classify people into the different categories (e.g. self-report, census or administrative data, social media data, etc.)* *Please provide details about how you controlled for confounding variables in your analyses.*
Population characteristics	*Describe the covariate-relevant population characteristics of the human research participants (e.g. age, genotypic information, past and current diagnosis and treatment categories). If you filled out the behavioural & social sciences study design questions and have nothing to add here, write "See above."*
Recruitment	*Describe how participants were recruited. Outline any potential self-selection bias or other biases that may be present and how these are likely to impact results.*
Ethics oversight	*Identify the organization(s) that approved the study protocol.*

Note that full information on the approval of the study protocol must also be provided in the manuscript.

Field-specific reporting

Please select the one below that is the best fit for your research. If you are not sure, read the appropriate sections before making your selection.

☐ Life sciences ☐ Behavioural & social sciences ☒ Ecological, evolutionary & environmental sciences

For a reference copy of the document with all sections, see nature.com/documents/nr-reporting-summary-flat.pdf

Ecological, evolutionary & environmental sciences study design

All studies must disclose on these points even when the disclosure is negative.

Study description	We conducted a meta-analysis of 12 infant cohorts across 8 countries to model gut virome development over the first three years of life. We identify the first year of life as the most dynamic period for virome development, in terms of richness and rate of maturation, and convergence of communities toward a stable, common, state by 3 years of age.
Research sample	The aim was to characterize infant gut virome development using publicly-available virome data from 12 previously-published studies, consisting of 1,893 samples from 999 infants across 8 different countries.
Sampling strategy	Sampling strategy was not considered as we used publicly-available virome data.
Data collection	Data collection is described in the methods, under the "Study curation" section.

Timing and spatial scale	This study analyzed 1,893 samples from 999 infants across 12 cohorts using publicly-available datasets. The infants were aged from 0 to 38 months. No additional experiments were performed.
Data exclusions	Following read mapping and the application of coverage detection thresholds (≥1X depth and ≥75% breadth of coverage), 1,869 samples from 991 infants were retained for downstream analysis (24 samples from 8 infants had insufficient sequencing depth for analysis).
Reproducibility	Our analyses can be reproduced using the code and information provided.
Randomization	Randomization was not considered as we used publicly-available virome data.
Blinding	Blinding was not considered as we used publicly-available virome data.

Did the study involve field work? ☐ Yes ☒ No

Reporting for specific materials, systems and methods

We require information from authors about some types of materials, experimental systems and methods used in many studies. Here, indicate whether each material, system or method listed is relevant to your study. If you are not sure if a list item applies to your research, read the appropriate section before selecting a response.

Materials & experimental systems

n/a	Involved in the study
☒	☐ Antibodies
☒	☐ Eukaryotic cell lines
☒	☐ Palaeontology and archaeology
☒	☐ Animals and other organisms
☒	☐ Clinical data
☒	☐ Dual use research of concern
☒	☐ Plants

Methods

n/a	Involved in the study
☒	☐ ChIP-seq
☒	☐ Flow cytometry
☒	☐ MRI-based neuroimaging

Plants

Seed stocks	*Report on the source of all seed stocks or other plant material used. If applicable, state the seed stock centre and catalogue number. If plant specimens were collected from the field, describe the collection location, date and sampling procedures.*
Novel plant genotypes	*Describe the methods by which all novel plant genotypes were produced. This includes those generated by transgenic approaches, gene editing, chemical/radiation-based mutagenesis and hybridization. For transgenic lines, describe the transformation method, the number of independent lines analyzed and the generation upon which experiments were performed. For gene-edited lines, describe the editor used, the endogenous sequence targeted for editing, the targeting guide RNA sequence (if applicable) and how the editor was applied.*
Authentication	*Describe any authentication procedures for each seed stock used or novel genotype generated. Describe any experiments used to assess the effect of a mutation and, where applicable, how potential secondary effects (e.g. second site T-DNA insertions, mosiacism, off-target gene editing) were examined.*
