# Supplementary material for: A meta-analysis of infant gut viromes reveals global patterns in bacteriophage community assembly and functional capacity over the first three years of life
Source: Nat Commun. 2026 Jun 18;17:7698. doi: 10.1038/s41467-026-74609-5 (PMC13434738; doi:10.1038/s41467-026-74609-5)
Supplement: Supplementary file 4 [file 41467_2026_74609_MOESM5_ESM.pdf]

nature portfolio

Peer Review File

A meta-analysis of infant gut virome reveals global patterns in bacteriophage community assembly and functional capacity over the first three years of life

Corresponding Author: Dr Corinne Maurice

This file contains all reviewer reports in order by version, followed by all author rebuttals in order by version.

Version 0:

Reviewer comments:

Reviewer #1

(Remarks to the Author)
Summary

In this work, the authors performed a meta-analysis of infant gut viromes utilizing sequencing data from 12 studies across the globe, specifically focusing on identifying global patterns in phage community assembly and functional capacity analysis. This is a large amount of sequencing data analyzed with multiple sites has the potential to be very informative. Limitations include lack of studies from the developing world, focus on DNA viromes. While viromes were individual, by using phage host families (PHF), the authors reported shared patterns by age group. Their regression model was able to accurately predict infant age from derivative sequencing data (vOTUs converted into PHF), and they authors present data regarding common virome functional changes across time. This approach of using PHF, however, seems to be assessing prevalence/presence of bacterial families as predicted by phage host rather than the actual virome. Thus the study seems more a description of the predicted bacteriome rather than the phageome without actual measurements of the bacteriome, and at lower resolution taxonomic level than if MGS for bacteriome analysis had been performed. I agree that limitations of viral taxonomic classification make broad generalizations of the virome challenging, but this approach focusing on the bacterial hosts de-emphasizes the viruses. The reasoning behind using this metric and how this metric differs from simple bacteriome analysis were not well presented or explained. This limits my enthusiasm for the manuscript.

Major Comments

1. Methods
a. How did the authors account for artifactual differences between studies (i.e. due to different processing methods, different sequencing machines, etc)? What negative or positive controls were available with each of these studies and how did they compare across the cohorts?

b. What was the minimum contig length kept for downstream analysis? Please list default SPAdes parameters.

c. It appears that the Family taxonomic level was used for diversity metrics. Why was genus or species level not chosen? Are there similarities by species level or do the similarities disappear at lower taxonomic levels?

2. Infant delivery mode, infant sex, human milk vs formula feeding, and antibiotics use (both infant and maternal) all have an impact on the infant gut microbiome/virome. The model should take these variables into account. If only a subset of samples have this data, please analyze the subset to ensure these factors are not skewing the results.

3. Lines 172-174: the authors report that developmental velocity was based on weighted UniFrac distance. Many (possibly even most) viruses under the new taxonomic classification have no well-defined phylogenetic tree. How were the authors able to use viral phylogeny and why did they choose this metric for the developmental velocity instead of Bray-Curtis or another metric that doesn't depend on how good or poor phylogenetic trees are? Or were the authors still using the PHF phylogeny rather than vOTUs? If PHF, please indicate within that paragraph and the reasoning behind using this. For a

virome paper, beta diversity metrics of the virome (vOTUs) would be preferred over the PHF.

4. Do the authors have bacteriome data? I am concerned that the virome development velocity, measured using PHF, may be simply reflecting bacteriome maturation rather than an actual metric of the viral maturation. It would be beneficial to show there are differences between the “viral maturation” and “bacterial maturation”. This work would further benefit from direct measurement of the bacteriome, to ascertain if these metrics are merely reflecting age differences in the underlying bacteriome.

5. How was library size accounted for (line 250)?

Minor Comments

1. The authors citation #21, Roux and Emerson 2022, for their statement in lines 59-60 does not support this statement. This 2022 review article focuses specifically on issues with soil viromes, and encourages use of both VLP and MGS as complementary ways to assess the virome, though no direct data is presented for this view either. Please amend or remove this statement to reflect that there is no data to support the authors claim, or provide another citation that has data to support it.

2. Similarly, line 62 is rather strongly worded without evidence to support this. I would posit that many of the intracellular, integrated viruses likely have metabolic genes actively in play and thus contributing the metabolomic milieu/functional capacity of the virome (per the authors the purpose of their paper as stated in line 59), which would be missed by assessing extracellular VLPs alone. Further, VLP methods are known to remove of some members of the virome (large viruses, viruses bound to blood protein components, etc). The best extraction method (VLP vs MGS) is an active area of debate within the field, with not much data to support either position, I would suggest rewording/toning down the definitive statements regarding this topic.

3. Please add average sample sequencing depth of each of the studies to Table S1

4. Please include in methods which 24 samples were not retained for downstream analysis (same study or different studies).

5. Was figure 2C based on vOTU richness/evenness/diversity or PHF richness/evenness/Shannon diversity?

(Remarks on code availability)

Reviewer #2

(Remarks to the Author)
This manuscript presents an in-depth analysis of infant gut virome development over the first three years of life using data pooled from 12 previously published studies. The authors take a highly innovative approach of examining both viral operational taxonomic units (vOTUs) as well as predicted phase host families (PHFs). The analysis found that the virome is most dynamic in the first year of life, with an increase in richness and fastest rates of change (“virome developmental velocity (VDV)”) in the first 6-8 months. The study also outlined specific changes to temperate phages and phage-encoded metabolic genes to provide functional insights to the observed ecological changes. As the authors highlight well throughout the manuscript, this study was designed to overcome several key limitations of existing virome research and has several important strengths described below. Accordingly, the data from this study are highly novel, yield important insights into virome development during infancy, and provide a study design template for future work. This approach may yield new insights into the role of gut virome in growth, immunity and disease, and could be applied to understand better virome dynamics in other biological compartments.

Key strengths:

First, most virome studies are small and there is a lack of standardized methods, which limits comparability across studies and restricts our ability to draw biological and clinical conclusions from virome data. In this analysis, data from 12 studies that all used a similar sample processing approach (virus-like particle enrichment to capture actively replicating viruses) were pooled to create a large sample size that offers greater statistical power than most individual existing studies. The raw sequence data from each study were also re-processed using a single bioinformatics pipeline to eliminate potential methodological biases. The data were also sourced from several global regions (US, Europe, China), which remains rare in the current literature.

Second, most virome studies classify viruses into vOTUs, which are highly individual-specific and can obscure functional similarities in the virome between individuals and across populations (virome composition is also highly dependent on geography). To reduce this individual specificity, the authors used a novel analytic approach to group viruses into PHFs—specifically, viruses with different sequences but shared bacterial hosts are grouped together, which better reflects their functional role within the virome. As a result, the authors were able to observe significant within- and between-individual virome convergence over time, confirming patterns that had been observed in earlier studies. Certain data were also presented using both vOTU and PHF classifications to emphasize how the variability in vOTUs can alter results.

Third, the manuscript presents rigorous and thoroughly explained statistical methods to evaluate virome ecology and model virome dynamics longitudinally, including tailoring individual model assumptions to the distributions of the underlying data. There are consistent explanations of how the observed virome data are expected to parallel bacteriome data (e.g., in temporal trends), despite understandably not having bacteriome data from the same study populations.

Suggestions for revision:

1. This analysis focuses solely on bacteriophages. While these comprise the majority of the infant gut virome, were any other types of viruses identified in the samples?
2. Similarly, given that much of the virome consists of unknown viruses, what proportion of the vOTUs (and/or PHFs) could not be classified in this analysis? This information should be added to the manuscript and/or discussed as a limitation in the discussion.
3. Though only 21% of samples had data for infant sex and delivery mode, the sample size with these variables is fairly large relative to other virome studies (N=401). Could the authors conduct an analysis among this subset to evaluate whether findings differ when accounting for these characteristics?
4. Feeding is an important determinant of the infant gut virome, and breastfeeding and weaning practices differ greatly between populations. Did any of the original studies include infant feeding data to assess how the age cutoffs for infant milestones matched the study data? If available, could this data be incorporated into the analyses as with the infant sex and delivery mode data? If data were not available, please include a comment in the discussion about this limitation. It would also be helpful to provide a reference supporting the infant developmental milestone ages, especially since it is unclear whether these milestones are known to be the same in all global regions included in this study (e.g., are infants in the US, Europe, and China all expected to be fully weaned by 12 months?).
5. The purpose of the machine learning model analysis could be further clarified and addressed in the discussion. Additionally, could the authors provide additional detail to clarify how the conclusion that “the ability to accurately predict infant age based on PHF-level community composition confirms that the gut virome follows a predictable successional pattern” (page 17, lines 343-344) was reached? It also appears that the model’s predictive ability declines substantially after 12 months as indicated by the test data results in Figure 3C. Could the authors add a comment on why the model’s predictive power declined in this period? We assume this is because once the virome stabilizes it is no longer associated with age.
6. The title seems to undersell the important findings and innovative approach. Consider updating the manuscript title to better emphasize the use of pooled data and the focus on bacteriophages. The following sentence on page 4, lines 57-58 is particularly apt: “A Meta-Analysis of Infant Gut Viromes to Identify Global Patterns in Bacteriophage Community Assembly and Functional Capacity”.

Minor comments for the authors to consider:

1. Are there any bacteriome data that could be cited to strengthen the comment that virome shifts mirror bacteriome shifts (discussion section page 20, lines 405-408).
2. How do the results of the pooled analysis compare to the results of the original individual longitudinal studies? This comparison would make an interesting comment in the discussion section.
3. In the table in Figure 2D, consider replacing analysis variable names with labels.

(Remarks on code availability)

Reviewer #3

(Remarks to the Author)
I co-reviewed this manuscript with one of the reviewers who provided the listed reports. This is part of the Nature Communications initiative to facilitate training in peer review and to provide appropriate recognition for Early Career Researchers who co-review manuscripts.

(Remarks on code availability)

Version 1:

Reviewer comments:

Reviewer #1

(Remarks to the Author)
The authors have sufficiently addressed my prior comments.

(Remarks on code availability)

Reviewer #2

(Remarks to the Author)
We appreciate the attention to detail the authors took in addressing our comments on this manuscript. The new data provided as supplemental information will be of interest to many readers working on the human Virome. The authors addressed our questions and concerns. We have no additional suggested revisions.

(Remarks on code availability)

Reviewer #3

(Remarks to the Author)
I co-reviewed this manuscript with one of the reviewers who provided the listed reports. This is part of the Nature Communications initiative to facilitate training in peer review and to provide appropriate recognition for Early Career Researchers who co-review manuscripts.

(Remarks on code availability)

Open Access This Peer Review File is licensed under a Creative Commons Attribution 4.0 International License, which permits use, sharing, adaptation, distribution and reproduction in any medium or format, as long as you give appropriate credit to the original author(s) and the source, provide a link to the Creative Commons license, and indicate if changes were made.
In cases where reviewers are anonymous, credit should be given to 'Anonymous Referee' and the source.
The images or other third party material in this Peer Review File are included in the article's Creative Commons license, unless indicated otherwise in a credit line to the material. If material is not included in the article's Creative Commons license and your intended use is not permitted by statutory regulation or exceeds the permitted use, you will need to obtain permission directly from the copyright holder.
To view a copy of this license, visit https://creativecommons.org/licenses/by/4.0/

REVIEWER COMMENTS

Reviewer #1 (Remarks to the Author)

Summary

In this work, the authors performed a meta-analysis of infant gut viromes utilizing sequencing data from 12 studies across the globe, specifically focusing on identifying global patterns in phage community assembly and functional capacity analysis. This is a large amount of sequencing data analyzed with multiple sites has the potential to be very informative. Limitations include lack of studies from the developing world, focus on DNA viromes. While viromes were individual, by using phage host families (PHF), the authors reported shared patterns by age group. Their regression model was able to accurately predict infant age from derivative sequencing data (vOTUs converted into PHF), and they authors present data regarding common virome functional changes across time. This approach of using PHF, however, seems to be assessing prevalence/presence of bacterial families as predicted by phage host rather than the actual virome. Thus the study seems more a description of the predicted bacteriome rather than the phageome without actual measurements of the bacteriome, and at lower resolution taxonomic level than if MGS for bacteriome analysis had been performed.

I agree that limitations of viral taxonomic classification make broad generalizations of the virome challenging, but this approach focusing on the bacterial hosts de-emphasizes the viruses. The reasoning behind using this metric and how this metric differs from simple bacteriome analysis were not well presented or explained. This limits my enthusiasm for the manuscript.

We thank the Reviewer for their constructive summary and for highlighting the potential impact of this large-scale meta-analysis. We agree that while PHFs are inherently linked to the bacterial landscape, our goal was not to provide a "predicted bacteriome", but rather to establish an ecological framework for identifying global successional patterns that are otherwise obscured by high viral sequence individuality. We would like to clarify the utility of PHFs and their distinction from a bacteriome analysis.

As demonstrated in **Figure 2A**, the infant gut virome is extremely individualized at the vOTU level. In cross-individual comparisons, vOTU-based distance metrics like Bray-Curtis can reach saturation (distances near 1.0) because infants, across or even within the same cohort, share few individual vOTUs. To mitigate this vOTU-level heterogeneity, vOTUs can be grouped according to the bacterial host they are predicted to infect, as described in our prior work (1). Under this framework, multiple distinct vOTUs are aggregated according to shared host targets, rather than shared sequence identity. Under this framework, multiple vOTUs are expected to have comparable impacts through shared host targeting and can thus be considered together when assessing virome structure. By collapsing vOTU-level variation into host-informed groupings, PHFs enable the identification of conserved ecological signals across individuals.

In our recent benchmarking study (1), PHFs significantly reduced intra- and inter-individual distances compared to vOTUs. This resulted in the first two axes of beta diversity ordinations explaining nearly four times more variance (39.3% vs. 11%) than vOTU-level analyses. This reduction in noise allowed for the detection of successional and disease-specific viral signatures that were otherwise masked at the nucleotide level.

Crucially, PHF profiles are not proxies for host prevalence or abundance, as phage and host abundances are frequently decoupled. A review of computational approaches to predict phage-host pairings suggests that only

9.5% of phages have abundance profiles that strongly correlate with their host species due to non-linear Kill-the-Winner dynamics, variable burst sizes, host-encoded phage defense systems, and phage lifestyle (2). Thus, PHFs provide a unique, functionally informed representation of the viral predatory landscape.

Rather than de-emphasizing viruses, we employed a dual-resolution approach when appropriate. In our initial submission, we conducted analyses both at the vOTU and PHF level for viral prevalence (**Figure 2A/2B**) and beta dispersion (**Figure 3B**). Additionally, alpha diversity (**Figure 2C**) and modelling of temperate phage relative abundance (**Figure 4A**) were originally conducted at the vOTU level. Finally, our AMG analysis integrates both PHFs with vOTUs to assess AMG enrichment (**Figure 5**). We use PHFs alone when considering high-level ecological questions, such as evaluating the predictable assembly of the gut virome (**Figure 3C/3D**).

In response to the Reviewer's comments, in this revised version we also calculate virome developmental velocity (VDV) (**Figure 3A**) at the vOTU level and present these findings alongside the PHF-level VDV calculations (from initial submission).

We believe that our approach, combining sequence-level specificity (vOTUs) with functional convergence (PHFs), provides the most comprehensive view of the global infant virome to date.

Major Comments

1. Methods

a. How did the authors account for artifactual differences between studies (i.e. due to different processing methods, different sequencing machines, etc)? What negative or positive controls were available with each of these studies and how did they compare across the cohorts?

We thank the Reviewer for their important question regarding potential batch effects and artifactual differences between studies. We addressed these concerns through a three-tiered approach involving methodological standardization, stringent filtering, and robust statistical modeling.

1. Unified bioinformatic processing
    a. To eliminate bioinformatic batch effects (differences caused by using different assembly or identification tools), we did not rely on the processed data from the original publications. Instead, we re-analyzed the raw sequencing data from all 12 cohorts using a single, harmonized pipeline. This ensured that all vOTUs were identified and quantified using identical criteria across every sample.
2. Statistical Accounting for study-level variation
    a. In all statistical models (e.g., **Figure 2**, **Figure 3**, **Figure 4**), we utilized Generalized Additive Mixed Models (GAMMs) with a factor smooth interaction term (s(age_months, study, bs = "fs")). This is a powerful statistical approach that allows each study to have its own unique intercept and its own non-linear successional trajectory. By explicitly modeling "study" in this way, we account for the combined artifactual variance of different extraction protocols, library preparations, and sequencing platforms (as summarized in the table below). The global developmental patterns we report are the signals that persist after this study-specific variation is accounted for.
3. Contamination management & negative controls
    a. Regarding negative controls, we summarize the approaches taken by the original studies in the table below. While raw negative control data were available for less than half of the includes several

(n=5/12 with negative controls, see table below), we instead addressed potential contamination as follows:

   i. We restricted all analyses to vOTUs ≥ 3 kb, to remove short, fragmented noise which may be associated with low-biomass contaminants.
   ii. All included studies included some form of virus-like particle enrichment (usually fecal supernatant filtration followed by nuclease treatment), providing a shared methodological baseline that reduces the background bacterial DNA.

b. A recent study (3) specifically characterized the negativeome (the viral component of negative controls) in early life by analyzing four datasets which are also included in our study (Liang 2020a, Garmaeva 2024, Maqsood 2019, and Shah 2023). The authors found the median abundance of contaminating sequences to be low (1%), with abundances exceeding 10% in only 11% of evaluated samples, and that contaminants were largely study-specific. Contaminant sequences also had lower completeness compared to viral sequences from biological samples.
   The fact that we identified a conserved developmental program across 12 independent studies using different reagents, cohorts, and sites suggests that our findings are driven by a true biological signal rather than study-specific contaminants.

Parts of the following table have also been added to **Supplementary Data 1**.

**Study**	**VLP enrichment**	**DNA extraction**	**Library preparation**	**Sequencing approach**	**Negative controls**
Lim 2015	0.45um filtration of fecal supernatant	Roche COBAS Ampliprep	GenomiPhi MDA (DNA viruses) → Illumina Nextera DNA lib. prep.	Illumina MiSeq	N/A
Zhao 2017	0.45um filtration of fecal supernatant, lysozyme & DNase	Roche COBAS Ampliprep	GenomiPhi MDA (DNA viruses) → Illumina Nextera DNA lib. prep.	Illumina MiSeq	N/A
McCann 2018	0.45um filtration of fecal supernatant, PEG precipitation, DNase	Phenol-chloroform & QIAGEN Blood and Tissue kit	GenomiPhi MDA (DNA viruses) → Illumina Nextera XT DNA lib. prep.	Illumina MiSeq	N/A
Maqsood 2019	0.45um filtration of fecal supernatant	Roche COBAS Ampliprep	GenomiPhi MDA (DNA viruses) → Illumina Nextera DNA lib. prep.	Illumina MiSeq	PBS reagent control (n=1)
Liang 2020a	0.2um filtration of fecal supernatant, 100 kDa concentration, DNase & RNase	QIAGEN AllPrep DNA/RNA Mini kit	GenomiPhi MDA (DNA viruses) → Illumina Nextera XT DNA lib. prep.	Illumina MiSeq & HiSeq	Reagent controls (n=8), tube controls (n=5), cow milk controls

					(n=3), formula milk controls (n=3)
Beller 2022	0.2um filtration of fecal supernatant	QIAGEN QIAquick PCR Purification kit	WTA2 amplification (DNA & RNA viruses) → Illumina Nextera XT DNA lib. prep.	Illumina NovaSeq 500	PBS reagent controls (n=4)
Walters 2023	0.45um filtration of fecal supernatant, DNase cocktail & RNase	QIAGEN lysis buffer & EZ1 Virus Mini Kit v2.0	Illumina Nextera Flex DNA lib. prep.	Illumina NovaSeq 6000	Nuclease-free water control (n=1)
Shah 2023	0.45um filtration of fecal supernatant, 50 kDa concentration, “universal nuclease”	QIAGEN QIAmp viral RNA mini kit	GenomiPhi MDA (DNA viruses) → Illumina Nextera XT DNA lib. prep.	Illumina HiSeq X	N/A
Garmaeva 2024	0.45um filtration of fecal supernatant, PEG precipitation, DNase & RNase	Phenol-chloroform & QIAGEN Blood and Tissue kit	NEBNext Ultra/Ultra II DNA lib. prep	Illumina HiSeq 2000	SM buffer reagent controls (n=4)
Pannaraj 2018	0.2um filtration of fecal supernatant, CsCl gradient, Amicon YM-100 column, DNase	QIAGEN UltraSens Virus kit	GenomiPhi MDA (DNA viruses) → Illumina Nextera XT DNA lib. prep.	Illumina MiniSeq	N/A
Yan 2021	0.2um filtration of fecal supernatant, DNase & RNase	TIANGEN TIANamp Virus DNA/RNA kit	Random primer amplification (TIANamp) → unknown lib. prep. kit	Illumina HiSeq 3000	N/A
Liang 2020b	0.2um filtration of fecal supernatant, 100 kDa concentration, DNase & RNase	QIAGEN AllPrep DNA/RNA Mini kit	GenomiPhi MDA (DNA viruses) → Illumina Nextera XT DNA lib. prep.	Illumina MiSeq & HiSeq	N/A

b. What was the minimum contig length kept for downstream analysis? Please list default SPAdes parameters.

All contigs assembled by metaSPAdes were included as input to geNomad. After geNomad, vOTU clustering was carried out using all contigs, and those ≥3kb were retained for downstream analysis (host assignment, diversity analyses, modelling, etc.). This has been clarified in the methods of the main text (lines 106-114). The metaSPAdes parameters we used for assembly have also been added to the methods section (lines 104-106).

c. It appears that the Family taxonomic level was used for diversity metrics. Why was genus or species level not chosen? Are there similarities by species level or do the similarities disappear at lower taxonomic levels?

The decision to use the phage host's family (PHF) in our ecological and diversity analyses was based on the technical accuracy of current host-prediction tools like iPHoP. In our recent benchmarking study (1), which evaluated the concordance of PHFs to experimental Hi-C data, we found that the family level represented the best balance between host taxonomic resolution and accuracy. The family level was the lowest taxonomic rank with the highest concordance (~93%) between computational predictions (iPHoP) and *in situ* approaches (Hi-C). At the genus level, concordance dropped to ~70%, while at the species level, iPHoP often yields multiple hits to different species within the same genus, introducing significant uncertainty that could confound large-scale meta-analysis results.

2. Infant delivery mode, infant sex, human milk vs formula feeding, and antibiotics use (both infant and maternal) all have an impact on the infant gut microbiome/virome. The model should take these variables into account. If only a subset of samples have this data, please analyze the subset to ensure these factors are not skewing the results.

We agree with the Reviewer that these variables are important for the gut microbiome and virome. As only a subset of studies included these data variables we initially didn't include in our global modeling analyses. We now analyzed the subset of samples with infant sex (n=774 samples; 41% of all samples), delivery mode (n=500; 26%), or diet metadata (n=645, 34%) by re-fitting all GAMMs to the corresponding sample subset using a formula which accounts for differences in these variables:

Model formula which includes sex metadata (fit to 774 samples):
RESPONSE ~ s(age_months, **by = sex**) + **sex** + log10(depth) + s(age_months, study, bs = "fs", m = 1) + s(subjectID, bs = "re")

Model formula which includes delivery mode metadata (fit to 500 samples):
RESPONSE ~ s(age_months, **by = delivery_mode**) + **delivery_mode** + log10(depth) + s(age_months, study, bs = "fs", m = 1) + s(subjectID, bs = "re")

Model formula which includes diet metadata (fit to 645 samples):
RESPONSE ~ s(age_months) + **diet** + log10(depth) + s(age_months, study, bs = "fs", m = 1) + s(subjectID, bs = "re")

In all the above formulae, *RESPONSE* is the response variable of interest: Pielou's evenness, Shannon diversity, richness, virome developmental velocity (VDV), beta dispersion (PHF or vOTU), or temperate phage relative abundance.

The results of our new analyses can be found in **Supplementary Figure 3** and described in the main text (lines 334-339; 362-366; 409-414; 458-466; 534-548). Briefly, the trends in alpha diversity, VDV, beta dispersion, and temperate phage relative abundance models were not skewed by inclusion of sex, delivery mode, or diet metadata. Although there were some significant differences in these metrics between male/female sexes, cesarian/vaginal birth modes, and diets, they were limited to specific timepoints or dietary comparisons. Thus overall, the trends we reported initially (increasing, decreasing, or stabilizing with age, depending on the metric) remained the same. These results demonstrate the robustness of the underlying data, where despite differences in sex, delivery mode, or diet, the gut virome develops similarly across all infants in our study.

Regarding antibiotics usage, only four studies (Yan 2021, Liang 2020b, Beller 2022, and Zhao2017) collected this metadata. None of the 10 infants in the Yan2021 study or the 11 infants in the Liang 2020b study were on antibiotics at time of sample collection. In total, only 5 samples were collected from infants actively on antibiotic (3 samples from the Zhao 2017 study, and 2 samples from the Beller 2022 study), a number which is too small to draw any meaningful conclusions from.

3. Lines 172-174: the authors report that developmental velocity was based on weighted UniFrac distance. Many (possibly even most) viruses under the new taxonomic classification have no well-defined phylogenetic tree. How were the authors able to use viral phylogeny and why did they choose this metric for the developmental velocity instead of Bray-Curtis or another metric that doesn't depend on how good or poor phylogenetic trees are? Or were the authors still using the PHF phylogeny rather than vOTUs? If PHF, please indicate within that paragraph and the reasoning behind using this. For a virome paper, beta diversity metrics of the virome (vOTUs) would be preferred over the PHF.

We thank the Reviewer for this suggestion. We have updated **Figure 3A** and the associated methods (lines 200-201) and results (lines 350-353) to include virome developmental velocity (VDV) calculated using both vOTU-level Bray-Curtis distances and PHF-level Weighted UniFrac distances.

As shown in the updated figure, both metrics reveal nearly identical trajectories, characterized by a high VDV at birth which decreases and stabilizes around 6 months of age. This consistency across taxonomic resolutions (vOTU vs PHF) and ecological distance metrics (non-phylogenetic vs. phylogenetic) demonstrates that the observed successional program is a robust feature of the infant gut virome that is not dependent on the specific computational approach employed.

4. Do the authors have bacteriome data? I am concerned that the virome development velocity, measured using PHF, may be simply reflecting bacteriome maturation rather than an actual metric of the viral maturation. It would be beneficial to show there are differences between the "viral maturation" and "bacterial maturation". This work would further benefit from direct measurement of the bacteriome, to ascertain if these metrics are merely reflecting age differences in the underlying bacteriome.

We agree with the Reviewer that the successional trajectories of the gut virome and bacteriome are intrinsically linked. However, we believe our data demonstrate that the virome developmental velocity (VDV) we report in our manuscript is a distinct viral signature and not a simple reflection of the bacteriome.

We observe the same significant VDV signal at the vOTU level as we do at the PHF level (see updated **Figure 3A**). Since vOTUs represent individual viral sequences, this turnover rate is measured independently of

bacterial host taxonomy. The consistency of this signal at the vOTU level confirms it is an inherent signature of virome development.

In addition, a unified, high-resolution meta-analysis of the bacteriome for these 12 cohorts is not feasible due to both data availability and technical heterogeneity. While all 1,893 samples in our study had VLP-enriched virome data available, several cohorts lack paired bacteriome data (Walters 2023, Liang 2020b) or did not make their datasets publicly available (Garmaeva 2024). Furthermore, among the studies that did sequence the bacteriome, there is extreme variation in methodology, ranging from 16S rRNA sequencing of different hypervariable regions (V1-V2, V4, V4-V5) to Ion Torrent and Illumina bulk metagenomics (see table below). While we successfully harmonized the virome data using a single VLP-based pipeline, the technical biases inherent in different 16S primers (4) and sequencing technologies (5) make a unified bacterial analysis of these datasets difficult to achieve.

Study	Bacteriome available?	Bacteriome sequencing approach
Lim 2015	Yes	16S (V4), Illumina
Zhao 2017	Yes	16S (V4), Illumina
McCann 2018	Yes	16S (V4-V5), Illumina
Maqsood 2019	Yes	16S (V4), Illumina
Liang 2020a	Yes	Bulk metagenome, Illumina
Beller 2022	Yes	16S (V4), Illumina
Walters 2023	No	-
Shah 2023	Yes	16S (V4), Illumina
Garmaeva 2024	Only upon request	Bulk metagenome, Illumina
Pannaraj 2018	Yes	16S (V1-V2), Ion Torrent
Yan 2021	Yes	Bulk metagenome, Illumina
Liang 2020b	No	-

Furthermore, not all studies included in our meta-analysis considered the bacteriome to the same extent, often evaluating only aspects relevant to their specific research questions.

Thus, we proceeded instead with a comparison to a recent global meta-analysis of 3,154 gut metagenomes from 1,827 healthy infants (6) to evaluate commonalities and differences in gut virome and bacteriome development. This meta-analysis serves as a robust alternative because it provides a high-resolution (± 3 months) model of 'microbiome age' across the first 18 months of life. By re-processing data from 12 countries through a uniform pipeline, the authors established universal developmental trajectories. Notably, four of these countries (Finland, Estonia, Ireland, United States) are also represented in our study and constitute nearly half of our analyzed samples.

While both meta-analyses successfully trained random forest models to predict infant age from bacteriome/virome composition, the primary predictive features identified by each model differ significantly.

The microbiome age model for the bacteriome from Fahur Bottino and colleagues relies heavily on the Shannon diversity (its third most important predictor) and increases in the *Lachnospiraceae* and *Ruminococcaceae* families. In contrast, our virome model identifies phages targeting *Ruminococcaceae*, *Rikenellaceae*, *Bacteroidaceae*, and *Bifidobacteriaceae* as primary predictors of age. We also find that virome Shannon diversity remains stable across the entire sampling period. Taken together, these distinct predictors and alpha-diversity trends demonstrate that while the virome and bacteriome develop in parallel, they follow distinct, decoupled, successional programs during early life.

We have updated the discussion to include this comparison with bacteriome development (lines 507-514).

5. How was library size accounted for (line 250)?

We clarified this sentence in the main text and added a reference to the Methods section for more information. All instances of "library size" have been replaced with "sequencing depth" for clarity and consistency throughout the manuscript.

Minor Comments

1. The authors citation #21, Roux and Emerson 2022, for their statement in lines 59-60 does not support this statement. This 2022 review article focuses specifically on issues with soil viromes, and encourages use of both VLP and MGS as complementary ways to assess the virome, though no direct data is presented for this view either. Please amend or remove this statement to reflect that there is no data to support the authors claim, or provide another citation that has data to support it.

2. Similarly, line 62 is rather strongly worded without evidence to support this. I would posit that many of the intracellular, integrated viruses likely have metabolic genes actively in play and thus contributing the metabolomic milieu/functional capacity of the virome (per the authors the purpose of their paper as stated in line 59), which would be missed by assessing extracellular VLPs alone. Further, VLP methods are known to remove of some members of the virome (large viruses, viruses bound to blood protein components, etc). The best extraction method (VLP vs MGS) is an active area of debate within the field, with not much data to support either position, I would suggest rewording/toning down the definitive statements regarding this topic.

We now cite Gregory et al. (2020), which demonstrates that while bulk metagenomics may provide higher overall detection rates for phages, the two methods capture largely distinct viral populations with only ~10% overlap. We have clarified that our study specifically targets the extracellular, free viral particle fraction. As noted by Gregory et al., VLP enrichment often yields longer viral contigs, which is critical for the high-resolution taxonomic (vOTU) and functional (PHF/AMG) analyses performed in our study (7). We have toned down the language on this topic, and instead frame our approach as a targeted ecological lens on the mobile, free virome.

3. Please add average sample sequencing depth of each of the studies to Table S1.

We have added the average sequencing depth of each sample to each of the studies in Table S1.

4. Please include in methods which 24 samples were not retained for downstream analysis (same study or different studies).

The 24 samples not retained for downstream analysis were from five different cohorts: Beller 2022 (n = 1), Liang 2020a (n = 8), Lim 2015 (n = 3), Maqsood 2019 (n = 1), and Walters 2023 (n = 11). We have updated the methods (lines 118-121) to include this information.

5. Was figure 2C based on vOTU richness/evenness/diversity or PHF richness/evenness/Shannon diversity?

Alpha diversity metrics (richness, Pielou's evenness, and Shannon diversity) are based on vOTUs. We highlight this in the main text (lines 128 and 325) and have further clarified this in the legend for **Figure 2C**.

Reviewer #2 (Remarks to the Author)

This manuscript presents an in-depth analysis of infant gut virome development over the first three years of life using data pooled from 12 previously published studies. The authors take a highly innovative approach of examining both viral operational taxonomic units (vOTUs) as well as predicted phase host families (PHFs). The analysis found that the virome is most dynamic in the first year of life, with an increase in richness and fastest rates of change ("virome developmental velocity (VDV)") in the first 6-8 months. The study also outlined specific changes to temperate phages and phage-encoded metabolic genes to provide functional insights to the observed ecological changes. As the authors highlight well throughout the manuscript, this study was designed to overcome several key limitations of existing virome research and has several important strengths described below. Accordingly, the data from this study are highly novel, yield important insights into virome development during infancy, and provide a study design template for future work. This approach may yield new insights into the role of gut virome in growth, immunity and disease, and could be applied to understand better virome dynamics in other biological compartments.

Key strengths:

First, most virome studies are small and there is a lack of standardized methods, which limits comparability across studies and restricts our ability to draw biological and clinical conclusions from virome data. In this analysis, data from 12 studies that all used a similar sample processing approach (virus-like particle enrichment to capture actively replicating viruses) were pooled to create a large sample size that offers greater statistical power than most individual existing studies. The raw sequence data from each study were also re-processed using a single bioinformatics pipeline to eliminate potential methodological biases. The data were also sourced from several global regions (US, Europe, China), which remains rare in the current literature.

Second, most virome studies classify viruses into vOTUs, which are highly individual-specific and can obscure functional similarities in the virome between individuals and across populations (virome composition is also highly dependent on geography). To reduce this individual specificity, the authors used a novel analytic approach to group viruses into PHFs—specifically, viruses with different sequences but shared bacterial hosts are grouped together, which better reflects their functional role within the virome. As a result, the authors were able to observe significant within- and between-individual virome convergence over time, confirming patterns that had been observed in earlier studies. Certain data were also presented using both vOTU and PHF classifications to emphasize how the variability in vOTUs can alter results.

Third, the manuscript presents rigorous and thoroughly explained statistical methods to evaluate virome ecology and model virome dynamics longitudinally, including tailoring individual model assumptions to the distributions of the underlying data. There are consistent explanations of how the observed virome data are expected to parallel bacteriome data (e.g., in temporal trends), despite understandably not having bacteriome data from the same study populations.

We thank the Reviewer for their positive feedback and have made the necessary changes and clarifications to the main text.

Suggestions for revision:

1. This analysis focuses solely on bacteriophages. While these comprise the majority of the infant gut virome, were any other types of viruses identified in the samples?

We thank the Reviewer for this important point regarding the non-bacteriophage fraction of the infant gut virome. While our study focuses on the successional dynamics of the phageome, we have now performed an additional characterization of non-phage viral sequences.

Using geNomad, we identified 534 vOTUs (1.1% of the total dataset) belonging to 31 non-phage viral families. While these viruses represented a small median fraction of the community (0.32%), their distribution was highly sporadic (mean = 7.4%, SD = 19.1%), likely reflecting transient dietary inputs, isolated infection events, or study-specific effects. We have added **Supplementary Figure 6** to summarize the prevalence and abundance of these non-phage viruses, and we have updated the results (lines 446-466) to reflect these findings.

2. Similarly, given that much of the virome consists of unknown viruses, what proportion of the vOTUs (and/or PHFs) could not be classified in this analysis? This information should be added to the manuscript and/or discussed as a limitation in the discussion.

We report that 36,800 of 49,745 vOTUs (74%) were assigned a host by iPHoP, consistent with (1). We have updated the results section of the manuscript to include this information (lines 309-310).

3. Though only 21% of samples had data for infant sex and delivery mode, the sample size with these variables is fairly large relative to other virome studies (N=401). Could the authors conduct an analysis among this subset to evaluate whether findings differ when accounting for these characteristics?

Please see response to question #2 from Reviewer #1. We have added the relevant new analyses in this revised version of the manuscript.

4. Feeding is an important determinant of the infant gut virome, and breastfeeding and weaning practices differ greatly between populations. Did any of the original studies include infant feeding data to assess how the age cutoffs for infant milestones matched the study data? If available, could this data be incorporated into the analyses as with the infant sex and delivery mode data? If data were not available, please include a comment in the discussion about this limitation. It would also be helpful to provide a reference supporting the infant developmental milestone ages, especially since it is unclear whether these milestones are known to be the same in all global regions included in this study (e.g., are infants in the US, Europe, and China all expected to be fully weaned by 12 months?).

We thank the Reviewer for this important comment. Of the 12 cohorts included, six provided information on feeding mode at the time of sampling (Beller 2022, Liang 2020a [164 of 184 samples], Maqsood 2019, Pannaraj 2018, Yan 2021, and Zhao 2017), representing 645 samples (34% of all samples).

To ensure our prevalence age bins (0, 1 to 3, 4 to 6, 7 to 12, and >12 months) align with actual dietary milestones, we visualized the distribution of feeding modes across these six cohorts (**Supplementary Figure 1**).

This analysis confirmed that the "transitional" bin (4 to 6 months) correctly captures the initial introduction of solid foods across all represented geographic regions.

We agree that the term "fully weaned" can be ambiguous across different global regions. Our age binning was intended to reflect the introduction of new dietary substrates which are important drivers of gut microbiome maturation.

While the total cessation of breastfeeding varies by individual and culture, the guidelines for the introduction of complementary foods are highly conserved across the regions included in this meta-analysis. In the United States, the American Association of Pediatrics (AAP) recommends the introduction of solids at approximately 6 months (8). The European Society for Paediatric Gastroenterology, Hepatology, and Nutrition (ESPGHAN) recommends that complementary foods be introduced between 4 and 6 months (9). The National Health Commission of the People's Republic of China (10) and the World Health Organization also have similar recommendations (11).

We have updated the methods section (lines 169-176) to replace "fully weaned" with "establishment of a diversified diet" and added the appropriate regional references to support these milestones.

We have also included diet metadata in our analyses, along with sex and delivery mode. Please see our response to question #2 from Reviewer #1 for details.

5. The purpose of the machine learning model analysis could be further clarified and addressed in the discussion. Additionally, could the authors provide additional detail to clarify how the conclusion that "the ability to accurately predict infant age based on PHF-level community composition confirms that the gut virome follows a predictable successional pattern" (page 17, lines 343-344) was reached? It also appears that the model's predictive ability declines substantially after 12 months as indicated by the test data results in Figure 3C. Could the authors add a comment on why the model's predictive power declined in this period? We assume this is because once the virome stabilizes it is no longer associated with age.

We have expanded upon the description of the random forest model in the results (lines 387-390) and discussion (lines 495-506) sections.

We clarified that the model's purpose was to determine if a conserved successional program exists within the infant gut virome across geographically diverse cohorts. By identifying PHFs that consistently predict age regardless of the cohort or country of origin, we define a baseline for healthy community maturation.

Regarding the conclusion that "the ability to accurately predict infant age based on PHF-level community composition confirms that the gut virome follows a predictable successional pattern", we clarified in the results (lines 387-390) that this conclusion was primarily supported by our leave-one-study-out cross-validation (LOSOCV), since a model trained on 11 studies can accurately predict the age of infants in a 12th (completely unseen) study.

Finally, regarding the model's loss in predictive power with age, we agree with the Reviewer in their assumption. We have added a comment to the discussion (lines 499-506) explaining that the increase in model error after 20 months likely reflects the stabilization of the virome as a whole.

6. The title seems to undersell the important findings and innovative approach. Consider updating the manuscript title to better emphasize the use of pooled data and the focus on bacteriophages. The following sentence on page 4, lines 57-58 is particularly apt: "A Meta-Analysis of Infant Gut Viromes to Identify Global Patterns in Bacteriophage Community Assembly and Functional Capacity".

We revised the title to better reflect our findings and their significance.

Minor comments for the authors to consider:

1. Are there any bacteriome data that could be cited to strengthen the comment that virome shifts mirror bacteriome shifts (discussion section page 20, lines 405-408).

As detailed in our Response to comment # 4 by Reviewer #1, we have updated the discussion (lines 507-514) to reference to a recent meta-analysis of 3,154 gut metagenomes. We compare and contrast the development of gut viral and bacterial communities and predictors of age.

2. How do the results of the pooled analysis compare to the results of the original individual longitudinal studies? This comparison would make an interesting comment in the discussion section.

We have updated the discussion section to contextualize our meta-analysis findings against the original cohorts, wherever possible (lines 476-477, 515-516, 538-541, 542-552). We highlight that our pooled results provide a high-powered consensus on successional hallmarks, including the initial expansion of viral richness and the age-dependent decrease in temperate phage relative abundance (observed in 4 and 3 constituent studies, respectively). Additionally, we confirm the high prevalence of eukaryotic viruses reported in 7 of the included cohorts. We identify a later divergence in Shannon diversity for cesarean section-born infants compared to vaginally-born infants (18-24 months in our study, versus 12 months in McCann et al.). Finally, we align our findings with established bacteriome maturation patterns in formula-fed infants.

3. In the table in Figure 2D, consider replacing analysis variable names with labels.

We have updated the figure accordingly.

Reviewer #3 (Remarks to the Author)

I co-reviewed this manuscript with one of the reviewers who provided the listed reports. This is part of the Nature Communications initiative to facilitate training in peer review and to provide appropriate recognition for Early Career Researchers who co-review manuscripts.

We thank the Reviewer for their consideration of our manuscript and feedback.

References

1. Shamash M, Sinha A, Maurice CF. 2025. Improving gut virome comparisons using predicted phage host information. mSystems 10:e01364-24.
2. Edwards RA, McNair K, Faust K, Raes J, Dutilh BE. 2016. Computational approaches to predict bacteriophage-host relationships. FEMS Microbiol Rev 40:258–272.
3. Kuzub N, Kurilshikov A, Zhernakova A, Garmaeva S. 2025. Negativeome characterization and decontamination in early-life virome studies. Nat Commun 16:6190.
4. Abellan-Schneyder I, Matchado MS, Reitmeier S, Sommer A, Sewald Z, Baumbach J, List M, Neuhaus K. 2021. Primer, Pipelines, Parameters: Issues in 16S rRNA Gene Sequencing. mSphere 6:e01202-20.
5. Durazzi F, Sala C, Castellani G, Manfreda G, Remondini D, De Cesare A. 2021. Comparison between 16S rRNA and shotgun sequencing data for the taxonomic characterization of the gut microbiota. Sci Rep 11:3030.
6. Fahur Bottino G, Bonham KS, Patel F, McCann S, Zieff M, Naspolini N, Ho D, Portlock T, Joos R, Midani FS, Schüroff P, Das A, Shennon I, Wilson BC, O'Sullivan JM, Britton RA, Murray DM, Kiely ME, Taddei CR, Beltrão-Braga PCB, Campos AC, Polanczyk GV, Huttenhower C, Donald KA, Klepac-Ceraj V. 2025. Early life microbial succession in the gut follows common patterns in humans across the globe. Nat Commun 16:660.
7. Gregory AC, Zablocki O, Zayed AA, Howell A, Bolduc B, Sullivan MB. 2020. The gut virome database reveals age-dependent patterns of virome diversity in the human gut. Cell Host Microbe 28:724–740.
8. Meek JY, Noble L. 2022. Technical Report: Breastfeeding and the Use of Human Milk. Pediatrics 150:e2022057989.
9. Fewtrell M, Bronsky J, Campoy C, Domellöf M, Embleton N, Fidler Mis N, Hojsak I, Hulst JM, Indrio F, Lapillonne A, Molgaard C. 2017. Complementary Feeding: A Position Paper by the European Society for Paediatric Gastroenterology, Hepatology, and Nutrition (ESPGHAN) Committee on Nutrition. J Pediatr Gastroenterol Nutr 64:119–132.
10. National Health Commission of the People's Republic of China. 2023. Nutrition Guidelines of Complementary Feeding for Infants and Toddlers (WS/T 678—2020). Biomed Environ Sci 36:899.

11. 2023. WHO Guideline for Complementary Feeding of Infants and Young Children 6-23 Months of Age1st ed. World Health Organization, Geneva.
